# Magnitude and Associated Factors of Herbal Medicine Use During Pregnancy Among Women Attending Antenatal Care in Public Health Institutions of Central Tigray, Northern Ethiopia (2020): Facility-Based Cross-Sectional Study

**DOI:** 10.1155/2024/9932851

**Published:** 2024-10-18

**Authors:** Hailemikael Gebrekidan, Gebregziabher Kidanemariam

**Affiliations:** ^1^Department of Nursing, College of Medicine and Health Science, Adigrat University, Adigrat, Ethiopia; ^2^Department of Midwifery, College of Health Science, Aksum University, Aksum, Ethiopia

**Keywords:** associated factor, herbal medicine use, northern Ethiopia, pregnant mother

## Abstract

**Introduction:** Herbal medicine is described by the World Health Organization (WHO) as plant-derived compounds, either raw or processed, that are thought to have therapeutic advantages. Herbal medication is becoming more and more popular worldwide, particularly during pregnancy. The WHO estimates that 80% of people utilize herbal remedies. In Ethiopia, grandmothers and grandfathers frequently use herbal medicine at home to treat common health issues. Instead of using medically proven treatments during their pregnancy, the majority of expectant mothers trust herbal remedies.

**Objective:** The aim of this study was to assess the magnitude and associated factors of herbal medicine use during pregnancy among women attending antenatal care in public health institutions of central Tigray, northern Ethiopia.

**Methods:** Facility-based cross-sectional study was conducted from March 01 to May 15, 2020. Data were collected using a structured and pretested questionnaire. Data were entered into Epi-data manager version 7.2.5 and analyzed using SPSS version 23. Both binary and multivariate logistic regression analyses were carried out to assess the associated factors. Variables with *p* value less the 0.2 in bivariate analysis were transferred to multivariate analysis, and variables with *p* value ≤ 0.05 were considered as significant.

**Result**: Out of the total 333 respondents, making a response rate of 100%, 176 (52.9%) used herbal medicine during current pregnancy. The most common medicines used during pregnancy were garlic (59.4%) and ginger (51.7%). Occupation that is housewife had educational status (AOR = 11.816, 95% CI 1.848–35.535), illiterate (AOR = 1.886, 95% CI 1.586–2.241), residency/rural (AOR = 2.905, 95% CI 1.173–7.197), and average monthly income less than 500 Ethiopian birrs (AOR = 7.621, 95% CI 2.691–21.585) were factors that are significantly associated with the use of herbal medicine during pregnancy.

**Conclusion and Recommendation:** Based on our results, herbal medicine use during pregnancy is highly practiced in this study, and occupation, residency, educational status, and average monthly income were the significantly associated factors of herbal medicine use during pregnancy. There is a need to educate and counsel pregnant women on the harmful effects of herbal medicine use during pregnancy by the health care personnel and health extension worker.

## 1. Introduction

Herbal medicine is defined by the World Health Organization (WHO) as plant-derived ingredients that are thought to have therapeutic benefits and that contain raw or processed components of one or more plants. Pregnancy is a condition linked to physiological changes that result in problems related to pregnancy, such as heartburn, nausea, vomiting, and constipation [1]. Across the world, the use of herbal medicine is steadily rising, particularly in pregnancy. Pregnant women in many rural parts of the world think that herbal remedies are “natural” and “safe” in comparison to contemporary pharmaceuticals. They also think that traditional medicine can treat medical conditions and enhance health during pregnancy, childbirth, and the postpartum period [2]. Although the usage of herbal remedies in pregnancy varies greatly between nations, many of the same plants are utilized; it is estimated that 80% of the population living in rural parts of poor countries rely on traditional medicine for their health requirements, including use during pregnancy [3]. In Ethiopia, grandparents and grandmothers often use herbal medicine at home to treat a variety of common health issues [4]. Instead of using treatments that have been scientifically proven to work, the majority of pregnant women trust herbal remedies [5].

In pregnancy, peppermint, ginger, thyme, chamomile, sage, aniseeds, fenugreek, green tea, and garlic are the most often used herbs to relieve nausea, vomiting, bloating, and stomach problems. Every trimester and pregnancy-related issues determined the appropriate timing for using herbs. Ginger has been used, for instance, to treat symptoms of the common cold and nausea and vomiting during the first trimester [3].

The conventional methods for preparing herbal remedies are not hygienic and can cause the uterus to contract, increasing the chance of an abortion [6]. Factors influencing the use of herbal medicine include sociodemographic traits, societal and cultural influences, perceived low costs of herbal goods, lack of access to the public health system, and the high expense of contemporary health care [7].

The use of herbal remedies during pregnancy increases the risk of morbidity and mortality for both the mother and the fetus, including reduced fetal survival, fetal discomfort, and early delivery [5]. Pregnant women's responses to conventional drugs and herbal medications, including unpleasant reactions like allergies, vary depending on whether they use naturally harmful herbal remedies or take too much of them [8]. Eighty-seven percent of pregnant women used herbal medicine in the third trimester primarily in an attempt to induce labor. However, the adverse effects vary depending on the trimester; congenital malformations can occur in the first trimester; and fetal distress, intrauterine growth retardation, and fetal hypoxia can occur in the second and third trimesters [9].

Pregnant women are frequently exposed to herbal preparations, frequently as a form of self-care; these items may have teratogenic effects on the developing fetus. Pregnant women are aware of the possible hazards associated with drug use during pregnancy, but many are unaware that herbal medications may potentially be hazardous. Many mistakenly felt that because something is natural, it must be harmless [10]. During the third trimester of pregnancy, pregnant women who were given either 800 mg/day of garlic pills or a placebo reported experiencing nausea for various causes related to the supplementation of garlic [11]. Plant species were frequently utilized as abortion treatments and to induce fetal loss; nearly half of the women who had an unsafe abortion had turned to traditional physicians [12].

Despite research shows that consuming dried ginger reduced nausea and vomiting [13], it may be mutagenic, antiplatelet, and abortifacient to both the mother and the fetus [14]. Pregnancy-related herbal medicine use is typically associated with higher rates of mother and fetal morbidity and death. The usage of natural medicines during pregnancy in Ethiopia's Tigray Regional State is not well understood. Therefore, the aim of this study was to assess the magnitude and associated factors of herbal medicine use during pregnancy. The study's outcome will assist in the provision of health education in prenatal care clinics. It also serves as a catalyst for the conduct of related research employing cutting-edge technique by medical professionals and other researchers as baseline data.

## 2. Methods

### 2.1. Study Design, Period, Area, and Study Populations

Institution-based cross-sectional study was carried out. The study was conducted from March 01 to May 15, 2020 (from recruitment to data collection), in the central zone of Tigray Regional State, in the northern part of Ethiopia, at a distance of 1024 km from Addis Ababa and 240 km far from the capital of Tigray. The pregnant women who volunteered for an interview were eligible for the study. The sources of participants were all pregnant women's attended antenatal care in governmental health facilities found in the central zone of Tigray, and the study participants were selected using systematic random sampling.

### 2.2. Eligibility Criteria

#### 2.2.1. Inclusion criteria

The study included all pregnant women who attended ANC follow-up at the central zone health facility during the data collection period.

#### 2.2.2. Exclusion criteria

Pregnant women who experienced serious illness during the time of data collection were not allowed to participate in the study.

### 2.3. Sample Size and Sampling Techniques

**Figure 1:**
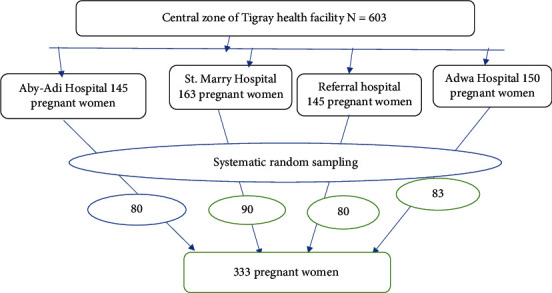
Schematic presentation of sampling procedure of magnitude and associated factors of herbal medicine use during pregnancy in governmental health facility in the central zone of Tigray.

The sample size was determined by using single population proportion formula using the following assumption: proportion of herbal medicine use among pregnant women (*p*) 73.1% [15], margin of error (*d*) = 5%, 95% confidence level, and 10% for possible nonresponse rate. It also assumed sample size calculation for different associated factors, so the final sample size was 333. During sampling, first simple random sampling was conducted to select health facility. Then, sample was taken from each selected health facilities proportional to population size using systematic random sampling technique to select study participants within each health institution (Figure 1).

### 2.4. Variables

The outcome variable is the use of herbal medicines during pregnancy which was operationalized as anything used in the promotion of health, prevention of illness, and treatment of diseases that are not prescribed by health care professional [16]. Some of the independent variables were age, educational level, marital status, ethnicity, religion, family size, parity, and occupation.

### 2.5. Operational Definition and Terms

• Urban residents: participants live in town
• Rural residents: participants live in rural
• Herbs: include crude plant materials such as leaves, flowers, fruit, seed, stems, wood, bark, roots, rhizomes, or other plant parts, which may be entire, fragmented or powdered.
• Herbal medicines: include herbs, herbal materials, herbal preparations, and finished herbal products that contain active ingredient parts of plants or other plant materials or combinations
• Use of herbal medicine: anything used in the promotion of health, prevention of illness, and treatment of diseases that are not prescribed by health care professional

### 2.6. Data Collection Tool

Primary data was collected from pregnant mothers who attended the selected health institutions using structured and pretested questionnaire. First, the questionnaire was prepared in English and translated to local language Tigrigna and translated back to English to observe its consistency. Finally, the questionnaire was pretested on 5% of pregnant mothers before actual data collection in nonsampled health facilities; correction and modification were done based on the gap identified during the interview. The questionnaire was grouped into three categories: sociodemographic characteristics, pregnancy-related factors, and status of herbal medicine use.

### 2.7. Data Collection Technique, Data Processing, and Analysis

Data were collected by face-to-face interview. The data was collected by four clinical midwives and supervised by the principal investigators. Two-day training was given to data collectors on the aim of the research, content of the questionnaire, and how to conduct interview to increase their performance in field activities. Daily supervision was conducted by the principal investigators, and the questionnaire was checked daily for completeness and consistency. Feedback was given to data collectors based on findings from the filled questionnaires.

Data were coded on prearranged coding sheet by the investigator, entered into Epi Info version 7.2.5, and exported to SPSS version 23 for analysis. Exploratory data analysis was done to check missing values, potential outliers, and normality distribution for those continuous variables. The presence of multicollinearity was checked by variance inflation factor, and in the study, variance inflation factor was 1.25. Model fitness was checked by Hosmer–Lemeshow goodness of fit statistics at 95%. Descriptive summaries of the study population were presented using frequencies and proportions.

Bivariate analysis was conducted to assess the crude association between dependent and independent variables. In the bivariate analysis, the independent variables with a *p* value less than 0.2 with the dependent variable were fitted into a multivariable logistic regression model to identify significant factors independently associated with the herbal medicine use. Finally, significant factors were identified based on AOR with 95% confidence interval. Those variables with *p* value less than 0.05 in the multivariable logistic regression analysis were considered as significant.

### 2.8. Limitation

Since the result depends only on the response of participants and retrospective nature, this study may have introduced recall bias which may have affected the estimated magnitude of herbal medicine use.

## 3. Results

### 3.1. Sociodemographic Characteristics

Initially, 333 participants were expected for interview, and finally, a total of 333 women were confirmed eligible in the study and making a response rate of 100%. The age of the respondents ranged from 18 to 45 years old with the mean age of 29 years (SD = ±5.9 years). Around 121 (36.3%) were in the age group of 24–29 years, 307 (92.1%) were married, 312 (93.7%) were Orthodox by religion, and 163 (48.9%) pregnant women were working as housewives (Table 1).

### 3.2. Pregnancy-Related Factors

Among the respondents, the majority (71.5%) were planned pregnancy. About 62.8% of the respondents were parity of one to two children. Approximately half (47.7%) of the respondents drawn in this study were in their third trimester of pregnancy. Of the total respondents, one hundred seventy-six (52.9%) had used herbal medicines during current pregnancy. Most of the respondents (56.9%) used the herbs in the first trimester of pregnancy (Table 2).

### 3.3. Factors Related to Herbal Medicine Use

**Figure 2:**
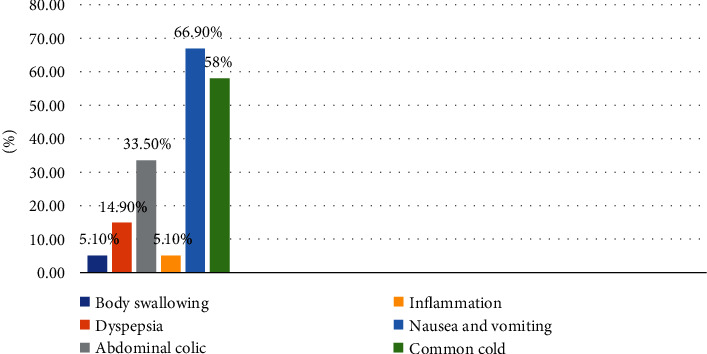
Indications of herbal medicine use in pregnant women attending ANC follow-up at public health institutions in the central zone of Tigray.

The most common reason for using herbal medicine was herbal medicines are accessible (67%) followed by safe in pregnancy (35.8%). The most common types of herbal medicines were garlic (59.4%) and ginger (51.7%). 96.6% of the herbal medicines were used oral. Nausea and vomiting 117 (66.9%) and common cold 102 (58.8%) were the common purpose to use herbal medicines (Figure 2). The most common source of information about herbal medicine was families, relatives, and friends (71.4%), followed by pregnant mothers who used herbal medicines (52.8%). The majority of the respondent who used herbal medicines (84.7%) did not discuss their use of herbal medicines with health care provider. The majority of herbal medicine users (70%) reported that they have not experienced any apparent side effects from herbs. Around half of the herbal medicine users were satisfied (47.7%) with the result of herbal medicine use. The most common side effect which was experienced by the users was burning sensation (75%). The most common reason for not using herbal medicine among nonusers was afraid of the side effects (46.5%) followed by the lack of belief in the benefits of herbs (35%) (Table 3).

### 3.4. Factors Associated With Herbal Medicine Uses

Binary logistic regression was done to assess association between independent variables and outcome variable with consideration of 95% confidence interval and *p* value of less than 0.05. Variables which were significant at binary logistic regression with *p* value of less than 0.2 were included in the multivariable logistic regression. At binary logistic regression, monthly income, educational status, occupation, residency, parity, trimester of pregnancy, and status of pregnancy were significant, but after multiple logistic regression, employed occupation, educational status, monthly income, and residency were significant. The occupation as housewife was 11.816 times more likely to use herbal medicine during pregnancy than governmental employee women (AOR = 11.816, 95% CI 1.848–35.535).

The odds of those participants with illiterates were1.886 times more likely to use herbal medicine during pregnancy than those who had diploma and above (AOR = 1.886, 95% CI 1.586–2.241). The result also revealed that rural residents were 2.905 times more likely to use herbal medicine for treatment purpose than the urban residents (AOR = 2.905, 95% CI 1.173–7.197). Respondents who had average monthly income of less than 500 ETB were 7.621 times much more risk for utilizing herbal medicine for treatment purpose than having income level of greater than 500 ETB (AOR = 7.621, 95% CI 2.691–21.585) (Table 4).

There was no missing value greater than 5% in all variable groups.

## 4. Discussion

In Ethiopia and other developing countries, as well as in this study area, herbal medicine use during pregnancy is a major health problem. Our study result shows that out of the total of 333 pregnant women, 176 (52.9%) and 99 (56.3%) had used herbal medicine during current pregnancy and during their first trimester, respectively.

Educational status, monthly income, employed occupation, and residency had significant association with herbal medicine use during pregnancy.

The magnitude of herbal medicine uses during current pregnancy among women attending antenatal care clinics at governmental health institutions in central zone was 52.9%. This result is consistent with the research conducted at Ethiopia's Gondar Referral Hospital (48.6%), [16] and Malaysia (51.4%) [9]. The similarity may be accessibility of the herbs.

However, the finding of this study was higher than the findings from Kenya 12% [17] and Gulu district, northern Uganda 20% [18]. It is lower than a report from southern Ethiopia which was 73.1% [15]. The difference in prevalence might be due to accessibility, affordability, and cultural issues regarding herbal.

Occupation was significantly associated with herbal medicine use among pregnant women; housewife was 11.816 times more likely to use herbal medicine during pregnancy than governmental employee women (AOR = 11.816, 95% CI 1.848–35.535). This finding is in line with the results of similar study conducted in Hosanna town, southern Ethiopia; this similarity might be due to similar socioeconomic status and methodology [15].

Herbal medicine use was the highest among those whose occupation was housewives; this is not in line with another study done in Kenya, Nigeria, and University of Gondar Ethiopia, where pregnant women who used herbal medicines for their pregnancy were the same as with other occupations in the previous study. The difference could be due to sociodemographic differences [7, 16, 17].

Educational level of the respondents was significantly associated with herbal medicine use. The odds of illiterate participants were 1.886 times more likely to use herbal medicine during pregnancy than those with diploma and above holders (AOR = 1.886, 95% CI 1.586–2.241). This is in line with the previous studies conducted in Ghana, Nigeria, Kenya, Gondar, and Hossana; this might be due to the fact that as educational level increases, the knowledge regarding modern medicine increases and attention is given for modern medicine than herbal medicines [15, 16, 19–21].

The result also revealed that rural residents were 2.905 times more likely to use herbal medicine for treatment purpose than the urban residents (AOR = 2.905, 95% CI 1.173–7.197). This is in parallel to another study done in Ghana [17]; Gondar referral hospital, Ethiopia [16]; and Hossana hospital [15] that herbal medicine was highly used in rural areas than urban areas. This is because rural residents have little knowledge about herbal medicine use and easily accessible.

Respondents who had average monthly income of less than 500 ETB were 7.621 times more to use herbal medicine for treatment purpose than having income level of greater than 500 ETB (AOR = 7.621, 95% CI 2.691–21.585), which in line with the study done in Gondar referral hospital revealed that the use of herbal medicine during pregnancy was significantly associated with average monthly income of the respondents; this might be due to lack of accessibility for modern medicines, as a result, they may rely on something accessible to them.

Here, monthly income, educational status, occupation, residency, parity, trimester of pregnancy, and status of pregnancy showed significant associations in the binary logistic regression, and finally, employed occupation, educational status, monthly income, and residency had a significant association with herbal medicine use during pregnancy.

## 5. Conclusion and Recommendations

The magnitude of herbal medicine uses during pregnancy in this study is high. Occupation, residence, average monthly income, and educational level were associated with herbal medicine uses during current pregnancy in this study. Out of the users, most of the respondents did not inform their health care provider. Therefore, behavioral change communication and information (BCC) is very important.

There is need to educate and counsel pregnant women on the harmful effects of herbal medicine use during pregnancy by the health care personnel and health extension worker.

## Figures and Tables

**Table 1:** Sociodemographic characteristics of pregnant women who attended ANC follow-up at selected public health institutions in central Tigray, Ethiopia (2020) (*N* = 333).

**Variables**	**Frequency (** **N** **)**	**Percentage (%)**
Age		
18–23	57	17.2
24–29	121	36.3
30–35	105	31.5
> 35	50	15
Marital status		
Unmarried	18	5.4
Married	307	92.2
Divorced	8	2.4
Religion		
Orthodox	312	93.7
Muslim	21	6.3
Occupational status		
Housewife	163	48.9
Private-owned business	62	18.6
Student	19	5.7
Governmental employee	81	24.3
Nongovernmental employee	8	2.4
Educational status		
Illiterate	91	27.3
Primary education (1–8)	71	21.3
Secondary education (9–12)	84	25.2
Diploma/degree	87	26.2
Residency		
Rural	124	37.2
Urban	209	62.8
Monthly income		
< 500	96	28.8
500–1300	42	12.6
> 1300	195	58.6

**Table 2:** Pregnancy-related factors among pregnant women who attended ANC follow-up at selected public health institutions in central zone of Tigray, Ethiopia (2020) (*N* = 333).

**Variables**	**Use of herbal medicine (%)**	**No use of herbal medicine (%)**	**Total (** **N** **) (%)**
Current pregnancy status			
Planned	115 (34.6)	123 (36.9)	238 (71.5)
Unplanned	61 (18.3)	34 (10.2)	95 (28.5)
Parity			
1–2 children	103 (30.9)	106 (31.8)	209 (62.8)
3–4 children	34 (10.2)	36 (10.8)	70 (21)
> 4 children	39 (11.7)	15 (4.5)	54 (16.2)
Trimester of pregnancy (GA)			
First trimester	21 (6.3)	32 (9.7)	53 (16)
Second trimester	67 (20.1)	54 (16.2)	121 (36.3)
Third trimester	88 (26.4)	71 (21.3)	159 (47.7)

Abbreviation: GA = gestational age.

**Table 3:** Herbal medicine uses during current pregnancy among antenatal care users in the health institutions of Central Zone, Tigray, Ethiopia (2020).

**Variables**	**Frequency (** **N** **)**	**Percentage (%)**
Herbal medicine uses during current pregnancy		
Yes	176	52.9
No	157	47.1
In which trimester they used the herbs (*n* = 176)		
First trimester	99	56.3
Second trimester	30	17
Third trimester	47	26.7
Reasons for used herbal medicines (*n* = 176)⁣^∗∗∗^		
Culturally	27	15.3
Belief on effectiveness	35	19.9
Herbal medicines are accessible	118	67
Treatment of other medical problems	46	26.1
Safe in pregnancy	63	35.8
Types of herbal medicines used (*n* = 176)⁣^∗∗∗^		
Ginger	91	51.7
Peppermint	31	17.7
Garlic	104	59.4
Tenaadam	77	44
Eucalyptus	54	30.9
Routes through which herbal medicines were used (*n* = 176)		
Oral	170	96.6
Smell	6	3.4
Sources of information to use (*n* = 176)⁣^∗∗∗^		
Families	125	71.4
Media (internet, television, radio, book)	9	5.1
Health care provider	6	3.4
Pregnant women who used herbal medicines	93	52.8
Have you experienced any effects after you used the herbal medicines (*n* = 176)		
Yes	51	29
No	126	70
If yes, what was your side effect (*n* = 51)		
Burning sensation	39	75
Vomiting	7	13.5
Dizziness	2	3.8
Malaise	2	3.8
Headache	2	3.8
Have you ever discussed using herbal medicines with your health care provider? (*n* = 176)		
Yes	27	15.3
No	149	84.7
In general, how could you rate the advantage you get from using herbal medicine use (*n* = 176)		
Satisfied	84	47.7
Neutral	43	24.4
Dissatisfied	49	27.8
If you have not used herbal medicine, why not (*n* = 156)		
Lack of belief in the benefits of herbs	55	35
Fear of side effect	73	46.5
Lack of availability	12	7.7
Did not get sick during gestation	17	10.8

⁣^∗∗∗^More than one option possible.

**Table 4:** Logistic regression variables on herbal medicine use for pregnancy in central zone of Tigray (2020) (*N* = 333).

**Variables**	**Use of herbal medicine**		**COR (95% CI)**	**AOR (95% CI)**
	**Yes (** **n** **)**	**No (** **n** **)**		
Occupation				
Housewife	146 (43.8%)	17 (5.1%)	14.314 (3.140, 25.245)	11.816 (1.848, 35.535)
Private-owned business	9 (2.7%)	53 (15.9%)	0.283 (0.057, 1.397)	0.165 (0.026, 1.067)
Student	12 (3.6%)	7 (2.1%)	2.857 (0.518–15.77)	1.701 (0.199, 14.562)
Nongovernmental	3 (0.9%)	5 (1.5%)	0.13 (0.25, 1.698)	0.077 (0.012, 1.505)
Governmental employee	6 (1.8%)	75 (22.5%)	1	1
Educational level				
Illiterate	73 (21.9%)	18 (5.4%)	18.066 (7.855, 23.549)	1.886 (1.586, 2.241)
Primary education	43 (12.9%)	28 (8.4%)	6.841 (3.047, 15.359)	1.457 (0.344, 6.177)
Secondary education	37 (12%)	47 (11.1%)	3.48 (1.506, 8.041)	1.437 (0.336, 6.177)
Diploma and above	23 (6.9%)	64 (19.2%)	1	1
Residency				
Rural	94 (28.2%)	30 (9%)	4.853 (2.955, 7.969)	2.905 (1.173, 7.197)
Urban	82 (24.6%)	127 (38.2%)	1	1
Monthly income				
< 500	79 (23.7%)	17 (5.1%)	8.486 (4.654, 15.472)	7.621 (2.691, 21.585)
500–1300	28 (8.4%)	14 (4.2%)	3.652 (1.804–7.395)	1.898 (0.593, 6.074)
> 1300	69 (20.7%)	126 (37.8%)	1	1

## Data Availability

All data are contained in the manuscript.
